# *Helichrysum italicum* ssp. *italicum* Infusion Promotes Fat Oxidation in Hepatocytes and Stimulates Energy Expenditure and Fat Oxidation after Acute Ingestion in Humans: A Pilot Study

**DOI:** 10.3390/plants10081516

**Published:** 2021-07-23

**Authors:** Saša Kenig, Katja Kramberger, Ana Petelin, Dunja Bandelj, Alenka Baruca Arbeiter, Ana Miklavčič Višnjevec, Kelly Peeters, Nina Mohorko, Karin Šik Novak, Zala Jenko Pražnikar

**Affiliations:** 1 Faculty of Health Sciences, University of Primorska, Polje 42, 6310 Izola, Slovenia; sasa.kenig@fvz.upr.si (S.K.); katja.kramberger@fvz.upr.si (K.K.); ana.petelin@fvz.upr.si (A.P.); nina.mohorko@fvz.upr.si (N.M.); karin.novak@fvz.upr.si (K.Š.N.); 2 Faculty of Mathematics, Natural Sciences and Information Technologies, University of Primorska, Glagoljaška 8, 6000 Koper, Slovenia; dunja.bandelj@upr.si (D.B.); alenka.arbeiter@upr.si (A.B.A.); ana.miklavcic@famnit.upr.si (A.M.V.); 3 InnoRenew CoE, Livade 6, 6310 Izola, Slovenia; kelly.peeters@innorenew.eu; 4 Andrej Marušič Institute, University of Primorska, Muzejski trg 2, 6000 Koper, Slovenia

**Keywords:** *Helichrysum italicum*, infusion, polyphenols, energy expenditure, fat oxidation, blood pressure, pilot study

## Abstract

*Helichrysum italicum* is an aromatic plant with promising pharmacological activities. Bioactive compounds found in plants represent an important alternative treatment for weight loss and an infusion of *H. italicum* contains compounds which could have such effect. Our aim was, therefore, to investigate its acute effects on resting energy expenditure (REE) and possible differences in substrate oxidation in a pilot study. A dried, ground plant material of *H. italicum* ssp. *italicum* was infused with hot water and chemically characterized with HPLC-MS analysis. Sensory evaluation of herbal tea was performed. A randomized, crossover, controlled pilot study was then conducted on eleven healthy male subjects. The REE and substrate oxidation were measured by indirect calorimetry at baseline and 30 and 120 min after ingestion of infusion or hot water. The expression of genes involved in lipid metabolism was examined in *H. italicum* infusion-treated hepatocytes. Several phenolic classes were identified in the infusion, caffeoylquinic acids were the most abundant, followed by pyrones and flavonols. A single ingestion of *H. italicum* infusion significantly increased REE by 4% and fat oxidation by 12% compared to hot water ingestion. A significant 2-fold up-regulation of β-oxidation-related genes in HepG2 cells, exposed to *H. italicum* infusion, was detected. This pilot study suggests that *H. italicum* infusion possesses bioactive substances with potential application in obesity prevention, which could, with additional studies, become an economically interesting novel application of the plant. Clinical trial registration number: NCT04818905

## 1. Introduction

*Helichrysum italicum* (Roth) G. Don (abbreviated as HI) is an aromatic dwarf shrub native to the Mediterranean region. Its plantations recently expanded mostly due to the demands of the cosmetic industry, where this plant is highly appreciated because of its characteristic scent. The plant is known as a rich source of biologically active compounds such as polyphenolic antioxidants, which are recognized in the prevention of various non-communicable chronic diseases [1]. This suggests that it could also be used in other economically interesting applications. In fact, it is traditionally used as an infusion or decoction to help with digestive, liver and gall disorders, inflammation and cough [2].

In our previous study by Kramberger et al. [3], the phytochemical profile of HI prepared with different extraction procedures was examined and one hundred compounds were identified, among which caffeoylquinic acids followed by pyrones and flavonols were the most abundant. Our key observation was that the hot water extracts (i.e., infusions) of *Helichrysum italicum* ssp. *italicum* (abbreviated as HII) proved to be comparably active as alcoholic ones, confirming the high commercial potential of HI preparations as herbal functional beverages in the category of phytotherapeutics. From a medicinal point of view, chlorogenic acid (CGA) and other related compounds such as isomers, derivatives and/or other quinic acid conjugates are potent antioxidants. Moreover, there is some evidence that CGAs have antihypertensive [4], anti-obesity, and anti-diabetic activity through positive effects on glucose and lipid metabolism and through the thermogenesis stimulation of brown adipocytes [5,6].

Obesity and overweight are global public health problems. In recent decades, rates of obesity in the world have increased at an alarming rate. According to the World Health Organization, an estimated 13% of the world’s adult population is currently obese. Obese and overweight individuals typically have elevated plasma levels of inflammatory markers and are subjected to increased oxidative stress; consequently, metabolic complications, insulin resistance, metabolic syndrome, type 2 diabetes mellitus, and cardiovascular disease have a much higher incidence in this population [7]. Low grade inflammation and oxidative stress can be successfully improved by appropriate lifestyle changes resulting in reduced body weight and body fat [8]. However, long-term maintenance of weight loss is often unsuccessful. Therefore, identifying strategies to increase energy expenditure (EE) and fat oxidation, or to decrease spontaneous energy intake (EI) may prove important in the management of body fat [9].

Bioactive compounds found in medicinal plants and plant extracts, such as polyphenols, represent the oldest and most widely used form of alternative or complementary treatments for the prevention and management of many noncommunicable diseases, among them also obesity. Their consumption is currently increasing in the population due to the high cost, potential adverse effects and limited benefits of currently available pharmaceutical drugs [10,11]. HI is a medicinal plant with promising pharmacological activities. Most of its traditionally claimed uses have not yet been scientifically proven [2], but the chemical profile of infusions suggests potential beneficial effects in obesity. Therefore, the aim of the present study was to investigate the acute effect of HII infusion on resting EE (REE), respiratory quotient (RQ) and blood pressure in a pilot study of 11 male adults. To further explore the effects of HII infusion on the lipid metabolism, additional in vitro experiments on hepatocytes were performed.

## 2. Results

### 2.1. Chemical Analysis and Sensory Evaluation of HII Infusion

Various phenolic classes were identified in HII infusion, among which hydroxycinnamic acids (HCAs), pyrones and flavonoids were the most abundant (Table 1). Although one dose of the active beverage contained several bioactive compounds, the majority belonged to the caffeoylquinic acids (CQAs) subclass.

**Figure 1:**
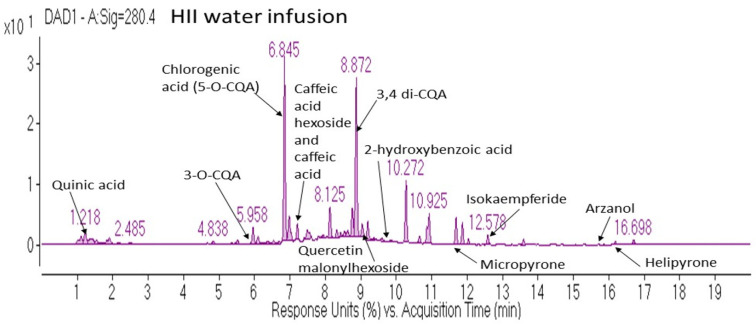
Representative diode array detector chromatogram at 280 nm of *H. italicum* ssp. *italicum* water infusion with peak annotations of the most characteristic compounds detected in the infusion. CQA—Caffeoyl quinic acid, DAD—diode array detector, HII—*Helichrysum italicum* ssp. *italicum*.

In Figure 1 are further presented some of the characteristic compounds identified in the HII infusion, representing compound classes mentioned in Table 1.

**Figure 2:**
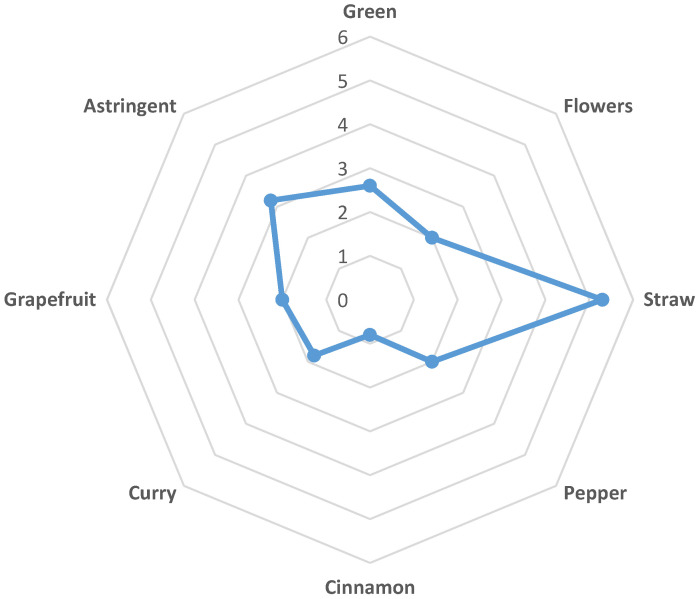
Sensory profile of HII infusion with possible aroma attributes is displayed as a spider web chart.

Twenty-nine tea consumers evaluated the likeability of the smell and taste, bitterness, and overall impression by hedonic tests from 1 (dislike very much) to 5 (like very much). Smell was evaluated as the most liked (Median 4; Range: 2–5) compared to the likeability of taste (Median 3; Range: 1–4), bitterness (Median 3; Range: 1–5) and overall impression (Median 3; Range: 2–4). Due to the notable astringency with the median value of 3.2, the prevalent likeability assessment of the infusion that amounted to 3 (neither like nor dislike) was expected. Moreover, the consumers that scored the bitter taste better, also assessed the likeability of the taste better (R = 0.4297; *p* < 0.05). Although the Spearman rank correlation was week between these two variables, it was significant. From the assessed attributes of the infusion (Figure 2), the aroma of straw was outstanding (Median 5.3). The aroma of green, flowers, some spices (curry and pepper) and of grapefruit was graded with approximately the same low intensity around 2. There was a slight cinnamon aroma detected with the median value intensity of 0.8. However, it must be pointed out that the possible aroma attributes of infusion were evaluated by untrained consumers and therefore, the variation of assessed scores ranging from 1.3 to 3.5 was high.

### 2.2. Baseline Characteristics of Study Participants

Baseline general characteristics of the male individuals are presented in Table 2. All participants had a normal body mass index (BMI), with fat percentage of 13.9 ± 3.1. Fat free mass of the individuals was 65.6 ± 9.1 kg on average. Systolic and diastolic blood pressures were 121 ± 8 and 74 ± 6 mmHg, respectively. No statistically significant differences in anthropometric parameters were observed between the two groups of the cross-over design, presented in Section 2.3.

**Figure 3:**
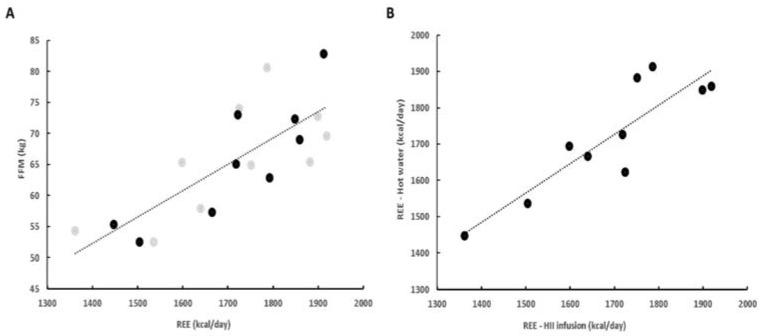
(**A**) Positive correlation between FFM and REE (R = 0.78; gray—HII infusion, black—hot water). (**B**) Positive correlation between initial REE before ingestion of HII infusion and hot water (R = 0.88). FFM—fat free mass; HII—*Helichrysum italicum* ssp. *italicum*; REE—resting energy expenditure.

Whole-body REE before the ingestion of beverages positively correlated with FFM (Figure 3A). Moreover, we observed a positive correlation between the baseline measurements before each ingestion for whole-body REE (R = 0.88, *p* < 0.001) (Figure 3B) and RQ (R = 0.77, *p* < 0.001), indicating a minimal intraindividual variation of REE and RQ and confirming the reproducibility of our measurements.

### 2.3. Acute Effects of a Single Ingestion of HII Infusion on Energy Expenditure and Blood Pressure

**Figure 4:**
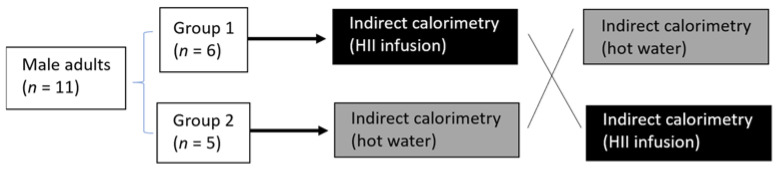
Study protocol for the acute pilot trial; randomized, controlled, crossover study.

To examine the acute effects of bioactive beverage in our setting, we first measured the response of whole-body REE to a single ingestion of either HII infusion or control beverage in the crossover design (Figure 4).

#### 2.3.1. Resting Energy Expenditure

**Figure 5:**
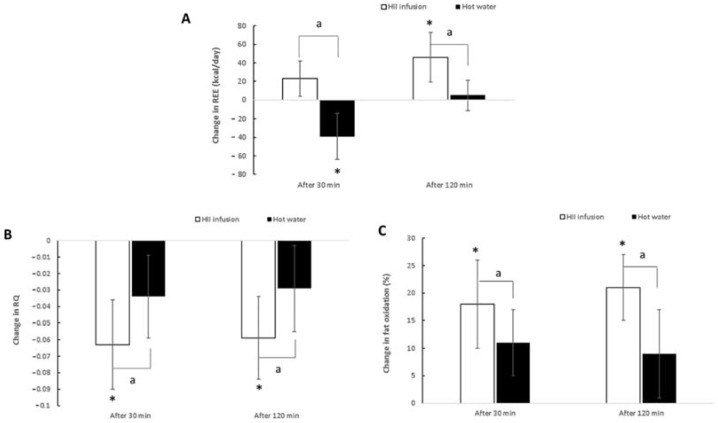
Changes in REE (**A**), RQ (**B**) and fat oxidation (**C**) after oral ingestion of HII infusion or hot water at 30 min and 120 min. Asterisk(s) indicate significant differences between post intake and baseline with * *p* < 0.05. Letter(s) indicates significant difference between changes in both trials with ^a^ *p* < 0.05. HII—*Helichrysum italicum* ssp. *italicum*; REE—resting energy expenditure; RQ—respiratory quotient.

A significant trial × time interaction was found for REE (*p* < 0.01) (Table 3). There were no significant differences at baseline between the bioactive and control trials (*p* = 0.86). However, REE was significantly higher 30 min after ingestion for the HII infusion compared to the control trial (*p* < 0.01) and the same at 120 min after ingestion (*p* < 0.01). Across time, REE increased at 30 min and even further at 120 min (*p* < 0.01) after taking the HII infusion compared to baseline. On the other hand, after the ingestion of the control drink, REE decreased significantly at 30 min (*p* < 0.01) and then returned to baseline. The HII infusion treatment induced a thermogenic response of 40 kcal per 2 h greater than hot water (Figure 5A). These results suggest that a single ingestion of HII infusion elicits a slight but significant increase in REE in healthy male adults.

#### 2.3.2. RQ and Fat Oxidation

Significant trial × time interactions were found for RQ and fat oxidation (*p* < 0.01 for both; Table 3, Figure 5B,C). At baseline, no significant difference was noted between HII infusion and control trials (*p* = 0.92 for RQ and *p* = 0.85 for fat oxidation). However, 30 min after ingestion, a significantly lower RQ and a significantly higher percentage of fat oxidation were observed for HII infusion compared to control (*p* < 0.01). At 120 min post-ingestion, RQ values remained significantly lower (*p* = 0.02) and percentage of fat oxidation was still significantly higher for HII infusion compared to control (*p* < 0.01). Compared to baseline, RQ and fat oxidation changed significantly 30 min after ingestion of HII infusion (*p* < 0.01) and remained significantly changed at 120 min post-ingestion. On the other hand, no significant changes across time were observed for the control trial (*p* = 0.11 for RQ and *p* = 0.09 for fat oxidation). These results suggest that a single ingestion of HII infusion elicits a significant increase in fat oxidation and, consequently, a significant decrease in RQ in healthy male adults.

#### 2.3.3. Blood Pressure

Systolic and diastolic blood pressures of the individuals following HII infusion consumption were also evaluated. Neither the HII infusion nor the control beverage influenced the SBP. A significant change was noted in DBP, which slightly declined at 30 min after HII infusion consumption (Table 3).

### 2.4. In Vitro Experiments in Hepatocytes

**Figure 6:**
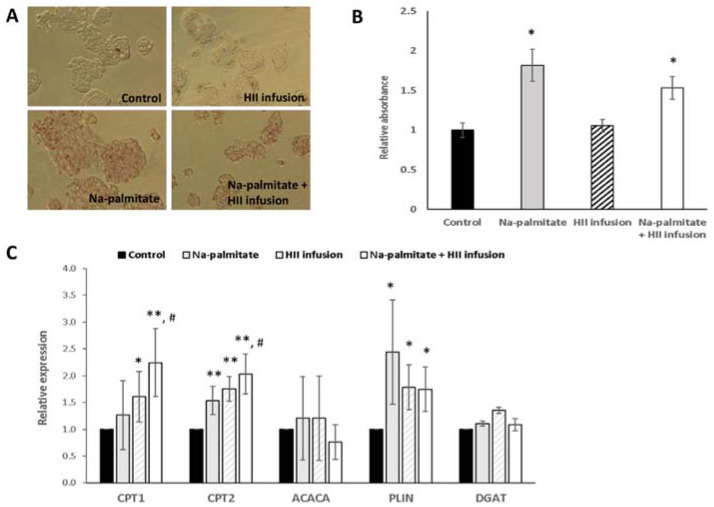
OilRedO staining (**A**,**B**) and relative gene expression (**C**) in HepG2 cells after the treatment with Na-palmitate, Na-palmitate and HII infusion, and HII infusion compared to control: (**A**) HepG2 cells stained with OilRedO after treatment; (**B**) Relative absorbance (mean ± SD); (**C**) Relative expression (mean ± SD) of different genes (CPT1, CPT2, ACACA, PLIN, DGAT). Asterisk(s) indicate significant differences compared to the control cells with * *p* < 0.05 and ** *p* < 0.01. Hashtags indicate significant differences compared to the Na-palmitate-treated cells with # *p* < 0.05. ACACA—acetyl-CoA carboxylase α; CPT1—carnitine palmitoyl-transferase 1; CPT2—carnitine palmitoyl-transferase 2; DGAT2—diacylglycerol O-acyltransferase 2; HII—*Helichrysum italicum* ssp. *italicum*; PLIN2— perilipin-2; SD—standard deviation.

Due to the observed changes in RQ and fat oxidation, we further investigated the effects of HII infusion on the metabolism of fats in vitro in HepG2 cells. OilRedO staining showed no effect of HII infusion alone on lipid droplet accumulation (Figure 6A,B). In cells treated with Na-palmitate, the absorbance at 517 nm was 1.82 ± 0.20-fold higher compared to control non-treated cells, showing that Na-palmitate treatment increased lipid accumulation in the control cells. The addition of HII infusion, which had no effect in control cells, did not prevent the Na-palmitate-induced lipid droplet accumulation. The increase was 1.57 ± 0.15-fold compared to non-treated cells but the difference in comparison to Na-palmitate alone was not significant (*p* = 0.09).

To explore the potential activity of HII infusion at the transcriptional level in hepatocytes, the expression of five genes, related to fat oxidation (CPT1 and CPT2), fatty acid synthesis (ACACA), triacylglycerol synthesis (DGAT) and lipid droplet formation (PLIN2) was determined by reverse transcription-polymerase chain reaction (RT-PCR) in HII infusion-treated cells compared to Na-palmitate and non-treated cells (Figure 6C). Treatment with Na-palmitate, Na-palmitate and HII infusion, and only with HII infusion upregulated fatty acid oxidation genes. Compared to the control, the expression of CPT1 was only slightly increased after the incubation with palmitate alone but was 2.3 ± 0.6-fold (*p* < 0.01) higher in cells treated with Na-palmitate and HII infusion. The upregulation of CPT2 gene was significant when cells were exposed to Na-palmitate (1.5 ± 0.2-fold, *p* < 0.01) and was further increased by the addition of HII infusion (2.0 ± 0.3-fold, *p* < 0.01). CPT1 is a mitochondrial enzyme responsible for the formation of acyl carnitines by catalyzing the transfer of the acyl group of a long-chain fatty acyl-CoA from coenzyme A to L-carnitine, while CPT2 catalyzes the reconjugation of long and very-long-chain acylcarnitines to coenzyme A following translocation into the mitochondrion. Both enzymes are important for fatty acid oxidation. On the other hand, HII infusion slightly downregulated ACACA, a gene that catalyzes the irreversible carboxylation of acetyl-CoA to produce malonyl-CoA and is therefore involved in fatty acid synthesis. Compared with the control, the expression of ACACA gene was decreased when HII infusion was added. The change was not significant (0.7 ± 0.3-fold, *p* = 0.19). HII infusion or Na-palmitate did not have any impact on DGAT. The reaction catalyzed by DGAT is considered the terminal and the only committed step in triglyceride synthesis and to be essential for intestinal absorption and adipose tissue formation. Upregulation of lipid droplet associated protein (PLIN2) was noted after the addition of Na-palmitate to HepG2 cells (2.4 ± 0.9-fold, *p* = 0.03), but was not as marked in cells exposed to Na-palmitate and HII infusion (1.7 ± 0.4-fold, *p* = 0.01).

## 3. Discussion

This is the first clinical study showing that consumption of HII infusion acutely increases fat oxidation and REE but has no significant effect on blood pressure. The induced metabolic effects found in the present study could be attributed to a number of bioactive components of HII infusion, hydroxycinnamic acid derivatives, arzanol derivatives and flavonoids and their interactions.

In various experimental protocols, CGA and its main metabolites such as ferulic acid, caffeic acid and isoferulic acid were detectable in the systemic circulation [12]. Farah et al. [13] demonstrated that over 30% of ingested cinnamic acid components were recovered in plasma, indicating that the major CGAs are highly absorbed and metabolized by humans. The remaining two-thirds are passed into the large intestine where the phenolic acid is further metabolized by gastrointestinal microflora and then absorbed [14].

Hydroxycinnamic acid derivatives, especially CGAs, have shown numerous health benefits in the treatment of obesity, cardiovascular disease, type 2 diabetes mellitus, and metabolic syndrome [15,16]. In the present study, the slight but significant effect was observed on REE, fat oxidation and RQ after a single intake of HII polyphenols. Ingestion of HII infusion increased REE by approximately 4% and fat oxidation by 12% for up to 2 h compared with hot water consumption. The present results are consistent with previous studies showing anti-obesity activity of CGA [17,18,19]. Soga et al. [18] showed that consumption of CGA led to a significantly higher postprandial energy expenditure compared to the control beverage. Thom [19] observed that consumption of instant coffee fortified with CGA significantly reduced body mass and body fat in overweight subjects. An effect of CGA consumption on BMI was observed by Watanabe et al. [20] in patients with mild hypertension, while Park et al. [17] showed that ingestion of CGA containing beverages for 5 days increased fat oxidation during sleep. Several mechanisms of action seem to be involved in the observed effect. However, the acute and chronic effects of CGAs should be discussed separately. It has been shown that CGA and the related hydroxycinnamic acid analogues inhibit cAMP phosphodiesterase, leading to an upregulation of adenosine monophosphate-activated protein kinase (AMPK), a key sensor of energy metabolism, with a consequent increase in fatty acid oxidation [21]. This mechanism supports our findings that a single ingestion of HII infusion led to increased REE and fat oxidation, after only 120 min. This time period is in agreement with Monteiro et al. [12], who reported the presence of unmetabolized CQAs in human plasma 2.3 h (T_max_) after acute ingestion of coffee containing CQAs. As noted by Stalmach et al. [14], most of the chlorogenic-derived compounds were rapidly removed from the circulatory system, with elimination half-life (T_1/2_) values of 0.3 to 1.9 h. The only unmetabolized compounds detected in plasma were three feruloylquinic acids and trace concentrations of 5-O-CQA. In our study, EE decreased 30 min after ingestion of hot water. This is different to the study of Boschmann et al. [22]; however, they also show that with the 22 °C water ingested, EE increased by 70 kJ and with 37 °C water, it only increased by 40 kJ. In our study, the temperature of both the HII infusion and the control drink was approximately 55 °C. The observed change in RQ after hot water ingestion was not significant in our study, but the trend was similar to that reported previously [22].

Since hepatic lipid metabolism plays a central role in whole-body lipid metabolism, we performed additional experiments on HepG2 hepatocytes to see whether the effects of HII infusion are only acute, or detectable also at the transcriptional level. We found that a 24 h treatment with HII upregulated the expression of CPT1 and CPT2 and downregulated ACACA suggesting increased β-oxidation. Despite this increase in gene expressions, the effect was not sufficient to lower lipid accumulation induced by Na-palmitate. The reduction in lipid accumulation caused by the addition of HII infusion to Na-palmitate- treated cells was not significant and neither was the concomitant reduction of PLIN expression. In contrast, Shimoda et al. [23] previously reported that in an animal model, green coffee bean extracts rich in CGA had an inhibitory effect on visceral fat accumulation and body weight gain. As for the mechanism, in agreement with our results, they reported that green coffee bean extract and phenolic compounds, such as neochlorogenic acid and feruloylquinic acid mixture, significantly increased hepatic CPT activity, which is a central component in fuel homeostasis. In agreement with Huang et al. [24], who found that CGA supplementation downregulated LXR-α mRNA expression with a corresponding decrease in ACACA, we also observed a decrease in ACACA expression, but it was not statistically significant. ACACA is an important rate-controlling enzyme in the synthesis of malonyl-CoA that inhibits CPT1. In other words, the decrease in ACACA leads to a decrease in malonyl-CoA content and a subsequent decrease in fatty acid synthesis and an increase in mitochondrial fatty acid oxidation through the regulation of CPT1. In addition to the acute effects, these results point to another possible mechanism through which HII infusions could exert their beneficial effects on fatty acid metabolism when consumed regularly.

HCAs may also improve cardiovascular health by exerting antihypertensive effects and acutely improving endothelial function [25]. Some studies suggest that chlorogenic acids can significantly lower systolic and diastolic blood pressures, while other studies instead report a lack of significant blood pressure reduction [20,26]. In the present study, the acute effect of HII infusion on blood pressure was not significantly different than control. However, it must be emphasized that we included only normotensive, healthy male subjects. Therefore, the different outcomes could be due to the fact that the blood pressure lowering effects of chlorogenic acids occurred only in subjects with mild hypertension [15]. Furthermore, because we only analyzed the acute effects of HII infusion in a short period of time (2 h), the hypotensive effect may not have been detected.

Several different classes of phenolic compounds were identified in the HII infusion; therefore, we cannot determine which bioactive molecule had an effect on the observed health benefits. However, chlorogenic acids as major constituents appear to be responsible for at least some observations. The second most abundant polyphenols in the HII infusion were arzanol and arzanol derivatives, which are characteristic compounds for HI. Arzanol was able to passively diffuse through the intestinal Caco-2 monolayers [27] and it is known to exhibit antioxidant and anti-inflammatory activities in biological systems [28]. However, the possible mechanism of arzanol as an anti-obesity compound remains to be elucidated. Flavonoids—mainly derivatives of quercetin and kaempferol, have also been detected in HII infusion. Quercetin is one of the most important flavonoids and one of the most potent antioxidants of plant origin. Although many in vitro studies and studies in animal models have focused on the beneficial effects of quercetin on obesity, there is only a limited number of human studies and clinical trials available [29]. One study evaluated the effects of quercetin intake in overweight subjects with various apolipoprotein E genotypes; quercetin (150 mg/day/subject) decreased waist circumference and plasma triacylglycerol concentrations [30].

The chemical profile of the HII infusion is complex and it is possible that more compounds have synergistic or additive effects. Studies on isolated compounds would be necessary to further explore the mechanism of action and identify the most active compound. However, from a phyto-therapeutic perspective, the administration of whole extracts or infusions is more appropriate than the ingestion of individual compounds to reduce the likelihood of the adverse effects’ occurrence [2]. Moreover, such product is also more readily available for the consumer, in particular in the Mediterranean regions where HI is often cultivated in plantations and even grown in home gardens. Considering previous reports showing different changes in EE after ingestion of water with different temperatures, the effect of cold infusions may be interesting to test.

The taste of infusions is also a very important factor contributing to the consumption and acceptance of the product among the consumers. Hedonic tests of HII infusions performed by unprofessional panelists demonstrated the acceptability of the smell, taste and overall impression. The prevalent aroma reported by the panelists was the aroma of straw and the bitter and stringent taste. According to Zhang et al. [31], tannins are one of the compounds contributing to the astringent taste and, in fact, they were detected in HII infusion [3]. The bitter taste detected by the panelists could be assigned to the caffeoylquinic acids, as Alasalvar et al. [32] in the study of carrots, suggested, that the chlorogenic acid could contribute to a mild bitter taste. However, Kreutzmann et al. [33] did not find the association between bitterness and phenolic acids including chlorogenic acids. Further studies should be done in order to relate the bitterness and astringency of this infusion with a chemical composition. In addition, to further assess the sensory profile of HII infusion, the involvement of a professional panel is necessary.

There are some limitations of our pilot study that should be mentioned. It included a relatively small number of participants, so further research with a larger number of subjects is needed to establish HII as a potential anti-obesity plant. As our measurements in human subjects were limited to 2 h after consumption, several questions about the effects of HII infusion remain unanswered. First, at what time point after a single dose does REE return to normal, do these effects diminish with continued ingestion (sensitization) and are these effects also observed in older adults and/or women. Therefore, further research should consider the effects of gender, age and chronic treatments on potential benefits for human health. Nonetheless, this in vivo pilot study along with the in vitro data, is the first study to demonstrate the effects of HI water infusion on the lipid metabolism, such as stimulation of REE and fat oxidation, both of which are beneficial mechanisms in the management of obesity. Taken together, our data suggests that further studies on HII are sensible.

## 4. Materials and Methods

### 4.1. Test Beverages’ Preparation and Chemical Analysis

Test beverages used in the present study were prepared from HII grown in commercial plantation in Dragonja, Slovenian Istria (45°27′05″ N 13°41′31″ E). Taxonomy classification and identity of HII plants was previously confirmed with the use of classical morphological characters according to Herrando-Moraria et al. [34] and by genotyping with microsatellite markers developed by Baruca-Arbeiter et al. [35]. Hot water extracts of HII (HII infusions) were prepared just before consumption by immersing tea filter paper filled with 1 g of milled dried plant material in hot water (200 mL, 100 °C) for 10 min. The control drink contained only hot water (200 mL, 100 °C); in addition, it also rested at room temperature for 10 min. Hot water was selected as a control drink based on similar studies [36,37,38] and because its influence on the RQ and EE has been investigated before [22].

Chemical composition of the HII infusion was analyzed by High performance liquid chromatography-mass spectrometry (HPLC-MS) analysis using an Agilent 1260 Infinity II HPLC system (Agilent Technologies, Santa Clara, CA, USA) equipped with a diode array detector (model G7115A) and coupled to an Agilent 6530 Accurate-Mass Quadrupole Time-of-Flight MS system equipped with an Agilent Jet Stream dual electrospray ionization source. The exact analysis conditions are described in Kramberger et al. [3]. The HII infusion was screened for the range of phenolic compounds in HI and identified, based on the accurate mass of precursor ions with minimum 80 overall match scores and fragmentation profile obtained from METLIN Metabolite and Chemical Entity Database (The Scripps Research Institute, San Diego, CA, USA) or literature data, if available.

Total phenolic content (TPC) was additionally determined using Folin–Ciocalteau reagent (FCR) in a microtiter plate protocol. The method was adapted from Magalhães et al. [39]. Briefly, 50 µL of HII infusion sample was mixed with 50 µL of FCR (1:5, *v/v*) in each well and 100 µL sodium hydroxide solution (0.35 M) was added. The reagent blank was evaluated by the addition of 50 µL of water instead of a sample. The absorbance was measured at 760 nm on the Multiskan SkyHigh Microplate Spectrophotometer (ThermoFisher Scientific, Waltham, MA, USA). TPC was calculated from the calibration curve for chlorogenic acid standard (Merck KGaA, Darmstadt, Germany) and expressed as mg of chlorogenic acid equivalents per g of dry mass. The estimated values of compound classes in Table 1 were quantified based on the determined TPC and relative abundance values of each identified compound. Three independent measurements with three replicates were performed.

### 4.2. Sensory Evaluation of Tea Samples

Hedonic tests of infusion samples were performed in order to evaluate the likeability of its taste, smell, bitterness, overall acceptability and the aroma, flavor and taste attributes. For this study, untrained consumers were recruited at the University of Primorska. Test beverage was prepared as described above. Recruited consumers were informed in advance that they would be evaluating a HII infusion with possible aroma, flavor and taste attributes such as straw, pepper, cinnamon, curry, grapefruit, etc. They were instructed to smell and drink the infusion. The recruited consumers were provided a prescribed questionnaire adopted from Adnan et al. [40] and Theron et al. [41] with appropriate adjustments. The sensory performance items in the prescribed questionnaire were 5: Like very much; 4: Like slightly; 3: Neither like nor dislike; 2 Dislike slightly; 1: Dislike very much. The aroma, flavor and taste attributes were rated from 1 (weakly intense) to 10 (very intense). The distribution of aroma, flavor and taste attributes is presented as a spider web chart (Figure 2).

### 4.3. Human Pilot Study

The protocol, approved by the National Ethics Committee (No. 0120-557/2017/4), was conducted in compliance with the Declaration of Helsinki and the International Conference on Harmonization Guidelines. In addition, the protocol for the present pilot study is registered on Clinicaltrials.gov (NCT04818905); however, the registration was conducted after recruitment of the first participant. To minimize within-subject variation, the study was designed to complete all measurements on a single subject within a week. Males were studied to avoid masking of the expected response by the variation in EE known to occur in premenopausal women during their menstrual cycle.

Subjects were recruited by word of mouth and posted announcements. Eleven apparently healthy male subjects agreed to participate in the study. They were weight-stable (±3 kg in the last 3 months), normotensive, non-smokers, had no use of dietary supplements nor frequent use of medication and had no known metabolic disorders. The subjects followed a normal Slovenian habitual diet and were asked to restrain from any intensive physical activity 12 h before the trial. After giving informed consent and being cleared for participation, the participants were randomly assigned to one of the 2 groups. Then their whole-body REE and its response to the ingestion of the HII infusion or control drink was measured in a randomized crossover design with indirect calorimetry (Figure 4).

All subjects completed both trials in a randomized, counterbalanced order on separate days at the same time of day (between 7 a.m. and 10 a.m.). Whole-body REE was measured with indirect calorimetry (Quark RMR, Cosmed, Rome, Italy). Prior to each testing session, volume and gas calibrations were performed following guidelines from the manufacturer. After overnight fasting for 10–12 h, the subjects relaxed on a bed in an air-conditioned room while wearing light clothing for >30 min under a thermoneutral condition at 25 °C. Then oxygen consumption (VO_2_) and carbon dioxide production (VCO_2_) were recorded for ~25 min by using a respiratory gas analyzer connected to a ventilated canopy hood. Subsequently, the subjects ingested a single dose of either the HII infusion or control drink within 2 min, and VO_2_ and VCO_2_ were again recorded for 2 h with breaks to avoid restraint stress. At 0, 30, and 120 min after ingestion, REE and RQ were calculated from the stable values of a 15 min period (the first 5 min were discarded for REE calculations).

Anthropometric variables were also measured on the day of the measurements. Subject height was measured to the nearest 0.1 cm in a standing position, without shoes, using a Leicester Height Measure (Invicta Plastics Limited, Oadby, UK). Body weight was measured with ±0.1 kg precision. BMI was calculated using the formula weight (kg)/height (m^2^). Body composition (total percentage body fat, total fat free mass (FFM) in kg, muscle mass in kg, total body water) was assessed by bioelectrical impedance analysis using a body composition analyzer Tanita BC 418MA (Tanita Corporation, Arlington Heights, IL, USA), and data were analyzed with the software provided by the producer. In addition, the same analysis also provided data on visceral fat rating. Heart rates, systolic blood pressure (SBP), and diastolic blood pressure (DBP) were measured during REE measurements (Omron M3, Omron, Japan).

### 4.4. In Vitro Experiments

Human liver cancer cell line HepG2 (ATCC^®^ HB-8056™) was purchased from ATCC^®^ (Manassas, VA, USA) and cultured in Dulbecco’s Modified Eagle Medium (DMEM) containing 1 g/L glucose, supplemented with 10% fetal bovine serum (FBS). Cell cultures were maintained at 37 °C in a humidified atmosphere containing 5% CO_2_. In serum-free medium, FBS was replaced by 1% bovine serum albumin (BSA).

HII infusion was freshly prepared as described above and diluted directly in cell culture media. For the experiments, 0.5% *v/v* infusion concentration was used, which was confirmed to be noncytotoxic for HepG2 cells using PrestoBlue™ Assay (Invitrogen™, Carlsbad, CA, USA). The Na-palmitate was first dissolved to 50 mM in 50% ethanol, sonicated, and then further diluted in serum-free DMEM containing 1% BSA, at 55 °C.

For the gene expression analysis, cells were plated to 6-well plates at a density of 300.000/well, left to adhere overnight and exposed to serum-free DMEM media containing 1% BSA for 24 h. Cells were then treated with HII infusions diluted to 0.5% *v/v* in serum-free cell culture media with or without 250 µM Na-palmitate (Sigma-Aldrich, St. Louis, MO, USA). RNA was isolated from infusion-treated and control cells using TRIzol^®^ reagent (ThermoFisher Scientific, Waltham, MA, USA) following manufacturer’s instructions. Two μg of RNA were reverse transcribed to cDNA with cDNA Archive kit (Applied Biosystems, Foster City, CA, USA) and the gene expressions were analyzed by QuantStudio^®^ 5 Real-Time PCR System (ThermoFisher Scientific, Waltham, MA, USA) using SYBR Green master mix and the following primer sequences: F-TCGCTTTGGGGGAAATAAATG and R-ACCACCTACGGATAGACCGC for acetyl-CoA carboxylase α (ACACA); F-TGGATCTGCTGTATATCCTTC and R-AATTGGTTTGATTCCTCCC for carnitine palmitoyl-transferase 1 (CPT1); F-AACCAACATGACTGTTTCTG and R-ATAGTGTCACTTTTTGCAGG for carnitine palmitoyl-transferase 2 (CPT2) [42]; F-CTG ATG AGTCCCACTGTGCTGA and R-TGTGGCACGTGGTCTGGAG for perilipin-2 (PLIN2), and F-GTGTGGCGCTACTTTCGAG and R-GTGGTCAGCAGGTTGTGTGT for diacylglycerol O-acyltransferase 2 (DGAT2) [43], 18S rRNA was used as an internal control (F-GTAACCCGTTGAACCCCATT and R-CCATCCAATCGGTAGTAGCG). Reaction conditions were 50 °C for 2 min, 95 °C for 10 min and 40 cycles of 95 °C for 15 s and 60 °C for 1 min. Melting curves were inspected to ensure primer specificity, the results were analyzed by the ΔΔCt algorithm and are presented as fold-changes compared to non-treated cells.

For the OilRedO staining, HepG2 cells were seeded on 6-well plates, left to adhere overnight, and starved in serum-free DMEM with 1% BSA for 24 h. They were then incubated in medium with 250 µM Na-palmitate with or without 0.5% *v/v* freshly prepared HII infusion. After 24 h of treatment, lipid droplets in HepG2 cells were revealed with an Oil Red staining kit from ScienCell Research Laboratories (Carlsbad, CA, USA) following the manufacturer’s protocol. The images were acquired using × 400 magnification. Previously stained cells were then exposed to 500 µL of isopropanol per well to solubilize the lipid droplets. Three aliquots of the obtained solution were transferred to a 96-well plate and the red coloration, proportional to the lipid droplet accumulation in the HepG2 cells, was measured spectrophotometrically (Multiskan Sky, ThermoFisher Scientific, Waltham, MA, USA) at 517 nm.

### 4.5. Statistical Analysis

Data were expressed as the means ± SEMs or as means ± SDs and analyzed using statistical software (SPSS 23.0; IBM, Tokyo, Japan). The Shapiro–Wilk test was used for the evaluation if the data distribution departs from the normal distribution. Comparisons between the 2 groups were analyzed using Student’s t-test or the Mann–Whitney U test, as appropriate. Changes in REE, fat oxidation, RQ, SBP and DSP from baseline were analyzed using the paired 2-tailed t-test or Wilcoxon’s Signed Rank test, as appropriate. Changes in REE, RQ and fat oxidation over 2 h after single ingestion of the bioactive or control drink were analyzed using 2-factor ANOVA for repeated measures on two within-subject factors (time and treatment) with post hoc multiple comparisons by Tukey’s post hoc test, as appropriate. Simple correlations were assessed using the Pearson correlation, or the Spearman rank correlation coefficient, as appropriate. A *p* value was considered statistically significant if ≤0.05. The data obtained in sensory evaluation of the infusion were analyzed using STATA13/SE software.

## 5. Conclusions

We have shown that a single consumption of HII infusion stimulates both REE and fat oxidation in normal weight men. This raises the possibility that HII infusion consumption could have some beneficial effects on an individual’s ability to maintain a lower body fat content. However, a positive effect would only be present if the effect persists with chronic consumption of HII infusion and the individual does not compensate by increasing food intake in response to taking HII infusion. Longer-term clinical trials would be necessary to further investigate our findings. Nonetheless, our results provide new insight into the health-promoting value of traditionally used HI infusions, which has been sparse before. Combined with the fact that the smell and taste of the infusion was found to be acceptable, the results could contribute to higher economic value of this widely grown plant.

## Figures and Tables

**Table 1:** Composition of the HII infusion. The estimated mean (M) ± standard deviation (SD), and minimum (Min) and maximum (Max) values of compound classes and subclasses (written in italic) were quantified based on the determined total phenolic content and relative abundance values of each identified compound. Three independent measurements with three replicates were performed.

	HII Infusion M ± SD (Min–Max)	Hot Water
Water (g)	200	200
Total identified phenolic compounds (mg)	15.5 ± 4.2 (10.0–20.0)	0
Total hydroxycinnamic acids (mg)	7.6 ± 1.3 (6.0–9.0)	0
*Caffeoylquinic acids* (mg)	3.0 ± 0.8 (2.0–4.0)	0
Total hydroxybenzoic acids (mg)	0.53 ± 0.17 (0.30–0.70)	0
Total flavonoids (mg)	1.4 ± 0.4 (1.0–2.0)	0
Total flavonols (mg)	0.7 ± 0.2 (0.5–0.9)	0
*Quercetin and its derivatives* (mg)	0.10 ± 0.05 (0.05–0.15)	0
*Kaempferol and its derivatives* (mg)	0.2 ± 0.1 (0.1–0.3)	0
Total arzanol derivatives and other pyrones (mg)	2.4 ± 0.9 (1.5–3.5)	0
*Arzanol* (mg)	0.2 ± 0.1 (0.1–0.3)	0

HII, *Helichrysum italicum* ssp. *italicum*.

**Table 2:** Characteristics of the participants included in the acute pilot trial.

	Mean ± SD All (*n* = 11)	Mean ± SD Group 1 (*n* = 6)	Mean ± SD Group 2 (*n* = 5)
Age (years)	34.7 ± 8.8	34.0 ± 8.7	35.2 ± 8.8
Height (cm)	182.8 ± 7.4	183.3 ± 6.1	181.8 ± 9.4
Weight (kg)	76.2 ± 10.6	74.5 ± 10.4	78.2 ± 10.2
BMI (kg/m^2^)	22.8 ± 2.1	22.3 ± 2.4	23.2 ± 1.6
Body fat (%)	13.9 ± 3.1	14.2 ± 2.7	13.8 ± 4.5
Fat mass (kg)	11.1 ± 3.1	11.2 ± 3.5	11.1 ± 4.3
FFM (kg)	65.6 ± 9.1	63.8 ± 8.7	68.3 ± 8.9
Blood pressure (mmHg)	121 ± 8/74 ± 6	122 ± 8/75 ± 7	119 ± 6/74 ± 3

BMI—body mass index; FFM—fat free mass; SD—standard deviation.

**Table 3:** The levels of REE, RQ, and hemodynamic parameters of 11 young men (mean ± SEM) at baseline, at 30 min and at 120 min post-intake.

	HII Infusion			Hot Water			Effect
	Baseline	30 min	120 min	Baseline	30 min	120 min	Trial × Time
REE (kcal/day)	1676 ± 56	1699 ± 45	1738 ± 42 *	1708 ± 61	1669 ± 65 *	1713 ± 62	<0.01
RQ	0.88 ± 0.02	0.82 ± 0.03 *	0.82 ± 0.02 *	0.87 ± 0.02	0.83 ± 0.02	0.84 ± 0.03	<0.01
Fat (%)	38 ± 8	56 ± 10 *	59 ± 6 *	41 ± 8	52 ± 9	50 ± 7	<0.01
SBP (mmHg)	120 ± 3	118 ± 3	120 ± 3	124 ± 3	121 ± 3	120 ± 3	0.345
DBP (mmHg)	74 ± 2	70 ± 2 *	71 ± 2	72 ± 3	71 ± 3	69 ± 3	0.657

DBP—diastolic blood pressure; HII—*Helichrysum italicum* ssp. *italicum;* REE—resting energy expenditure; RQ—respiratory quotient; SBP—systolic blood pressure; SEM— standard error of the mean. Asterisk(s) indicate significant differences from baseline with * *p* < 0.05.

## Data Availability

All data generated or analyzed during this study are included in this article.
